# Supplementary material for: Coronary embolism in left-sided infective endocarditis. A retrospective analysis from a high-volume surgical centre and review of the literature
Source: PLoS One. 2024 Dec 4;19(12):e0314718. doi: 10.1371/journal.pone.0314718 (PMC11616836; doi:10.1371/journal.pone.0314718)
Supplement: Attachment — Submitted filename: Response to Reviewers.docx [file pone.0314718.s001.docx]

Florence, November 11, 2024

Ahmed Qasim Mohammed Alhatemi

Academic Editor

PLOS ONE

Dear Dr Ahmed Qasim Mohammed Alhatemi

We are grateful for the opportunity to resubmit our paper entitled **“Coronary embolism in left-sided infective endocarditis. A retrospective analysis from a high-volume surgical center and review of the literature”** in revised form.

Each of the points raised by the reviewers has been carefully addressed, as detailed in the attached rebuttal letter. All authors have read and approved the final version of the paper.

We hope that, in its present form, our paper is now suitable for publication in “PLOS ONE”.

Looking forward to hearing from you, I send you my best regards.

Matteo Orlandi, MD

**Responses to Reviewer 1**

**Q1**. Being a retrospective review, is there any chance that some coronary embolization were not diagnosed because they did not result in clear ACS? was there any biomarker data that may shed more light on this point?

**R1**. We thank the reviewer for this comment. All patients with infective endocarditis included in our study were routinely screened with ECG and echocardiogram during their hospitalization. So, even if we cannot exclude that coronary embolization could have passed asymptomatic, we think that the proportion of patients correctly diagnosed with coronary embolization because of signs and symptoms of ischemia significantly outweighs the number of cases not diagnosed. Moreover, the vast majority of patients with left-sided infective endocarditis were treated surgically in our center and, thus, were screened with CT or invasive coronary angiography before surgery. Myocardial enzymes were drawn in all patients treated surgically, but we have to consider that acute myocardial injury is very frequent in the post-operative course and therefore cardiac enzymes values were not specific for the diagnosis of coronary embolization. After your suggestion, we further clarified this point in the “Limitations” section of our manuscript.

**Q2**. How systematic was the search for cerebral embolization, again could many cerebral emboli have been missed?

**R2**. In our center all patients with left-sided infective endocarditis are routinely screened with brain CT to search for potential cerebral emboli. Therefore, we think that only a slight minority of cerebral emboli could have been missed. In patients with signs or symptoms of cerebral ischemia, repeated CT scans or MRI scans were performed, reducing the possibility of missing the diagnosis of cerebral embolization.

**Q3**. The cases document when the coronary events occurred and I think it would be useful to include the time from presentation to the occurrence of coronary embolism in hours and also the duration of antibiotic therapy in tables 1 and 2 to see if embolic events represent delays in initiating antimicrobial therapy in relation to the diagnosis of possible infective endocarditis and try to understand better the predictors of coronary embolization.

**R3**. We thank the reviewer for this comment. Given the fact that our center is a third-level hospital for the treatment of infective endocarditis, especially because of the presence of in-site cardiac surgery facilities, many patients were transferred from other hospitals after the diagnosis of infective endocarditis was made and when antibiotic therapy was already started. Unfortunately, data regarding the time from presentation to the occurrence of coronary embolism and the duration of antibiotic therapy cannot be retrieved from our electronic charts. We agree with the reviewer that including these data could have shed new insights on the knowledge of the predictors of coronary embolization. This could be the focus of future studies.

**Q4**. It is not clear from the manuscript whether vegetation length was by 2D or 3D. This needs to be clarified.

**R4**. Vegetation length was measured with 2D transesophageal echocardiography.

**Q5**. Also I did not see any specific analysis for Coronary Embolization related to the mitral valve.

**R5**. We thank the reviewer for this comment. In our study only patients with left-sided infective endocarditis were included. We reported in Table 1 the rates of aortic valve infective endocarditis (56.5%) and aortic valve involvement among patients with coronary embolism (62.5%). Mitral valve infective endocarditis was present in 43.5% of patients and mitral valve was involved in 37.5% of patients with coronary embolism. The association between coronary embolism and aortic or mitral valve infective endocarditis was not statistically significant (p= 0.86). Involvement of anterior or posterior mitral leaflet, presence of mitral stenosis or mitral regurgitation and grading of valve disease were not parameters included in our dataset and therefore no specific analysis in terms of the relationship with coronary embolism can be done.

**Q6**. Is there any other echo parameter of the vegetation that can be related to cerebral or coronary embolization.

**R6**. In our analysis we did not find a significant association between vegetation length and the risk of coronary embolization. Other echo parameter, such as vegetation mobility and site of attachment, especially in the case of the mitral valve, could be potentially associated to cerebral or coronary embolization. However, these data were not routinely collected and therefore, in our study, no statistical analysis could be performed.

**Q7**. A limitation section need to be included.

**R7**. We thank the reviewer for the suggestion. We added a “Limitations” section to our manuscript.

**Response to Reviewer 2**

Thank you for this case series. I would like the authors to clarify how they decided to deal with the coronary emboli. In some cases, they left it alone and in some cases, they intervened. On what basis was the decision made? I also want to know if they analyzed the recovered embolic material and identified the causative pathogen, especially in culture negative cases. Finally, in case no.3, the authors state that the empirical antibiotics used were vancomycin and ceftriaxone, this is not a regular combination, why was it used?

**R**. As we outlined in the discussion, there is no clear evidence of the best strategy of treatment for patients with infective endocarditis and coronary embolization. In the majority of cases presenting with acute coronary syndrome, after the diagnosis of coronary embolization thromboaspiration was performed (case #1, #4, #6). In one case (case #5), coronary artery bypass graft was preferred. In one case (case #2) no treatment was performed, because of the distal localization of the occlusion. The patient of the case #7 died before that coronary angiography could be performed. In case #3 and #8, the diagnosis of coronary embolization was made after the results of the MRI, so that an interventional procedure could not be performed. In only one case (case #6) the embolic material was retrieved, and cultural exams tested positive for the same bacteria identified with blood cultures. We recognize that routine cultural exams on embolic material could have helped in the diagnosis of patient with negative cultures (case #2). In case #3, empirical antibiotic therapy with vancomycin and ceftriaxone was started. We recognize that this combination therapy is not usual, but the patient was transferred from another center after this combination of antibiotics was started. Treatment was later shifted to ceftriaxone and gentamicin after that Gemella morbillorum was found on the valve samples.
